# Flexible Strain Sensor Based on TPU/CNTs/rGO Conductive Membrane with Dual-Microcrack Structure for High-Performance Wearable Sensing

**DOI:** 10.3390/nano16191234

**Published:** 2026-09-30

**Authors:** Jinlong Chen, Binghao Xing, Yuexin Wang, Huimin Guo, Chenrui Zhu, Weihua Wang, Junfang Shen, Zhaowu Wang, Pengbo Zhao, Qian Sun, Derong Zhu, Zhiyu Min, Wei Zhai

**Affiliations:** 1 School of Intelligent Manufacturing, Luoyang Institute of Science and Technology, Luoyang 471023, China; jlchen@lit.edu.cn (J.C.);; 2 Henan Key Laboratory of Green Building Materials Manufacturing and Intelligent Equipment, Luoyang Institute of Science and Technology, Luoyang 471023, China; 3 Henan Mechanical and Electrical Vocational College, Zhengzhou 451192, China; 4 Key Laboratory of Materials Processing and Mold of Ministry of Education, School of Materials Science and Engineering, Zhengzhou University, Zhengzhou 450001, China

**Keywords:** flexible strain sensor, dual-microcrack structure, wearable electronics

## Abstract

Flexible strain sensors are crucial for wearable electronics and human–machine interaction, yet simultaneously achieving high sensitivity and a broad working range remains challenging. Here, a high-performance strain sensor is developed by integrating carbon nanotubes (CNTs)/reduced graphene oxide (rGO) hybrid conductive networks with electrospun thermoplastic polyurethane (TPU) nanofibers, followed by pre-stretching to engineer a dual-microcrack architecture. The synergistic interplay between 1D CNTs and 2D rGO stabilizes conductive pathways, while strain-regulated microcrack evolution amplifies resistance variation. The sensor delivers a wide strain range of up to 100%, with gauge factors of 75.2, 1600.5, and 7484.5 across 0–32%, 32–60%, and 60–100% strain, respectively. It further exhibits a rapid response/recovery time of 30/40 ms, a low detection limit of 0.01%, and excellent durability over 1000 cycles. Benefiting from these comprehensive superior sensing performances, the sensor can reliably monitor both subtle human physiological signals and large-amplitude human motions, demonstrating great application potential in wearable health monitoring, intelligent motion perception and human–machine interaction fields.

## 1. Introduction

In recent years, flexible strain sensors have emerged as promising platforms for wearable electronics, owing to their simple architectures, low cost, and tunable sensing characteristics, with growing applications in human health monitoring [1,2,3], human–machine interaction [4,5,6] and structure monitoring [7,8,9]. With the rapid development of intelligent and wearable systems, however, increasingly stringent requirements have been imposed on strain sensors, particularly in terms of sensitivity, sensing range, response speed, and operational stability. These performance metrics are often strongly coupled with the intrinsic physicochemical properties of flexible substrates and conductive functional materials, making it challenging to simultaneously achieve high sensitivity and a broad sensing range [10]. Accordingly, considerable efforts have been devoted to developing advanced flexible strain sensors through rational materials engineering and structural design to overcome these intrinsic performance trade-offs [11,12,13].

The selection and integration of conductive functional fillers represent a critical factor governing the electromechanical response of flexible strain sensors. Conductive networks based on zero-dimensional fillers, such as carbon black (CB) [14,15], and metal nanoparticles [16,17], as well as two-dimensional materials, including MXene [18,19], and reduced graphene oxide (rGO) [20,21], are generally susceptible to conductive pathway disruption under tensile deformation. Although the resulting rapid resistance variation is favorable for high sensitivity, it is often accompanied by a limited working range. In contrast, one-dimensional conductive fillers, such as carbon nanotubes (CNTs) [22,23], and metal nanowires [24,25] possess high aspect ratios and can form interconnected, deformable networks through entanglement and overlapping, thereby maintaining conductive connectivity under substantial deformation and enabling broader strain detection. For example, Wang et al. [26] sequentially deposited CNTs and silver nanowires (AgNWs) onto electrospun nanofiber membranes to construct breathable strain sensors with a bilayer conductive network. The synergistic interaction between the two 1D conductive nanomaterials enabled a strain-sensing range of up to 150%, a gauge factor (GF) of 1477.7, and stable operation over 7000 stretching–releasing cycles. In another study, Zhang et al. [27] fabricated eco-friendly strain sensors by vacuum-filtering graphene oxide (GO) onto silk fibroin nanofiber substrates followed by in situ reduction. The optimized sensor exhibited high sensitivity toward both tensile and bending deformation, with GFs of 436 and 204 at strains of 8–8.7% and 0.12%, respectively, enabling the detection of subtle physiological signals such as vocal vibrations and pulse waves. These studies demonstrate that tailoring the dimensionality and interaction of conductive fillers provides an effective route toward regulating the sensitivity and deformation tolerance of flexible sensors.

Beyond material selection, structural engineering of conductive networks offers another powerful strategy for regulating strain-transfer and signal-transduction behavior. Amorphous polymer matrices possess structural imperfections that generate band-edge disorder and gap-localized electronic states, which participate in charge transport within polymer–nanocarbon composites via variable-range hopping [28,29,30]. In particular, crack-based architectures can introduce highly localized changes in conductive connectivity, thereby generating pronounced resistance variation under small deformation and enhancing sensitivity. Wrinkled structures, by contrast, provide additional deformation accommodation through reversible geometric unfolding, while porous architectures combine low density, large specific surface area, and high deformability, making them attractive for strain-sensing applications [31,32]. By coupling these structural features, the electromechanical response of conductive networks can be further tailored across different strain regimes. For instance, Ji et al. [33] developed flexible strain sensors featuring hierarchical V-shaped grooves and wrinkled architectures using mold transfer, pre-stretching, and releasing processes. At low strain, the wrinkles effectively accommodated deformation and preserved conductive connectivity, enabling a sensing range of up to 45%. At higher strain, stress concentration at the V-shaped grooves promoted controlled crack propagation, resulting in an enhanced GF of 1557.71 while maintaining stable performance over 10,000 stretching–releasing cycles. Similarly, Zhou et al. [34]. fabricated crack-based strain sensors by spraying CNT ink onto electrospun nanofiber membranes followed by pre-stretching and releasing. The resulting microcrack network generated pronounced resistance variation even under subtle deformation, achieving GFs of 428.5 below 100% strain, 9268.8 within 100–220% strain, and 83,982.8 within 220–300% strain. These results highlight the effectiveness of combining conductive network engineering with strain-localization structures for enhancing sensor sensitivity over broad deformation regimes. Despite these advances, simultaneously integrating high sensitivity, broad sensing range, and structural stability within a single flexible strain sensor remains challenging. In particular, conventional strategies typically rely on either highly deformable conductive networks to extend the sensing range or unstable conductive pathways to amplify sensitivity, making it difficult to independently regulate signal amplification and mechanical tolerance. A rational strategy that synergistically couples conductive filler dimensionality with controllable crack evolution is therefore highly desirable. Beyond the optimization of individual sensing performance, the practical deployment of flexible sensors depends on system-level integration and multi-channel signal acquisition. Recent studies have verified the feasibility of constructing intelligent sensing platforms based on functional nanocomposite sensors, including integrated WO_3_/CNTs sensing systems and dual-mode MWCNTs-InSe monitoring devices [35,36]. Such advances demonstrate that flexible nanocarbon sensors can serve as basic units for arrayed sensing and intelligent monitoring systems.

Herein, we develop a high-performance flexible strain sensor by integrating one-dimensional CNTs and two-dimensional rGO with a deliberately engineered dual-microcrack conductive architecture. Flexible TPU nanofiber membranes are first fabricated by electrospinning to provide a mechanically compliant and breathable substrate. CNTs/rGO hybrid conductive ink is subsequently deposited onto the nanofiber membrane through spray coating, followed by a controlled pre-stretching–releasing process to generate a unique dual-microcrack network. The complementary structural characteristics of 1D CNTs and 2D rGO, together with the strain-dependent evolution of the dual-microcrack architecture, enable efficient regulation of conductive pathway connectivity under deformation. The morphology, electromechanical response, and sensing mechanism of the resulting TPU/CNTs/rGO sensor are systematically investigated. The sensor further demonstrates reliable detection of both subtle physiological signals and large-scale human motions, highlighting its potential for future array integration and wearable intelligent monitoring systems. This work provides a rational materials–structure co-design strategy for developing flexible strain sensors with simultaneously enhanced sensitivity, sensing range, and operational stability.

## 2. Experimental

### 2.1. Materials

Thermoplastic polyurethane (TPU, 1185A) was purchased from BASF SE (Ludwigshafen, Germany). Carbon nanotube (CNT) ink with an outer diameter of 5–10 nm was obtained from Suzhou Tanfeng Graphene Technology Co., Ltd. (Suzhou, China). Reduced graphene oxide (rGO, TNRGO151201) was purchased from Chengdu Organic Chemicals Co., Ltd. (Chengdu, China). Tetrahydrofuran (THF, analytical grade) was supplied by Tianjin Fuyu Fine Chemical Co., Ltd. (Tianjin, China), and N, N-dimethylformamide (DMF) was obtained from Tianjin Kemiou Chemical Reagent Co., Ltd. (Tianjin, China). All chemicals were used as received without further purification.

### 2.2. Preparation of Electrospinning Film

THF (12 mL) and DMF (12 mL) were mixed in a beaker and magnetically stirred for 10 min to obtain a homogeneous solvent mixture. Subsequently, TPU pellets (5.8 g) were added to the mixed solvent and stirred at 30 °C for 4 h until a homogeneous and transparent electrospinning solution was obtained. The solution was then transferred into a 10 mL syringe for electrospinning. The applied voltage and tip-to-collector distance were set to 21 kV and 18 cm, respectively. Under these conditions, uniform TPU nanofiber membranes were prepared by electrospinning.

### 2.3. Preparation of the Cnts/rgo Conductive Ink

CNT ink (50 mL) was first transferred into a beaker, followed by the addition of rGO (30 mg). The mixture was magnetically stirred at 30 °C for 60 min and subsequently subjected to ultrasonic treatment for 60 min to obtain a homogeneous CNTs/rGO hybrid conductive ink suitable for spray coating.

### 2.4. Preparation of Micro-Cracked Cnts/rgo/tpu Flexible Strain Sensor (CCRTS)

The CNTs/rGO hybrid conductive ink was spray-coated onto one surface of the electrospun TPU nanofiber membrane using a high-pressure airbrush (Yilong YL-130 airbrush, manufactured by Yiwu Yilong Tattoo Equipment Co., Ltd., Yiwu, Zhejiang, China). The airbrush-to-substrate distance was maintained at 10 cm, and the air pressure was set to 4 MPa. After spray coating, the samples were dried at 60 °C for 10 min. The same spray-coating and drying procedures were subsequently applied to the opposite surface of the TPU membrane to obtain a CNTs/rGO-coated TPU nanofiber membrane. Finally, the conductive membranes were subjected to controlled pre-stretching and subsequent release to generate strain-responsive microcrack structures, yielding the CNTs/rGO/TPU flexible strain sensor (CCRTS).

### 2.5. Characterization

The surface and cross-sectional morphologies of the CCRTS were characterized using scanning electron microscopy (SEM, JSW-7500F, JEOL, Tokyo, Japan). The electromechanical sensing performance was evaluated using a universal testing machine (UTM2203, Shenzhen Suns Technology Co., Ltd., Shenzhen, China) coupled with a high-precision digital multimeter (DMM4050, Tektronix, Beaverton, OR, USA). The resistance variation of the CCRTS was recorded simultaneously during mechanical deformation. For sensing-performance characterization, the digital multimeter was operated at a sampling rate of 1000 Hz. Response time is defined as the time for $\Delta R/R_{0}$ rising from 10% to 90% of peak signal upon step-strain loading; recovery time denotes the duration for $\Delta R/R_{0}$ dropping from 90% to 10% of peak signal after unloading. Step strain of 0.1% was applied at a cross-head speed of 1000 mm/min for response-recovery measurement.

## 3. Results and Discussion

### 3.1. Preparation and Morphological Characterization of CCRTS

**Figure 1:**
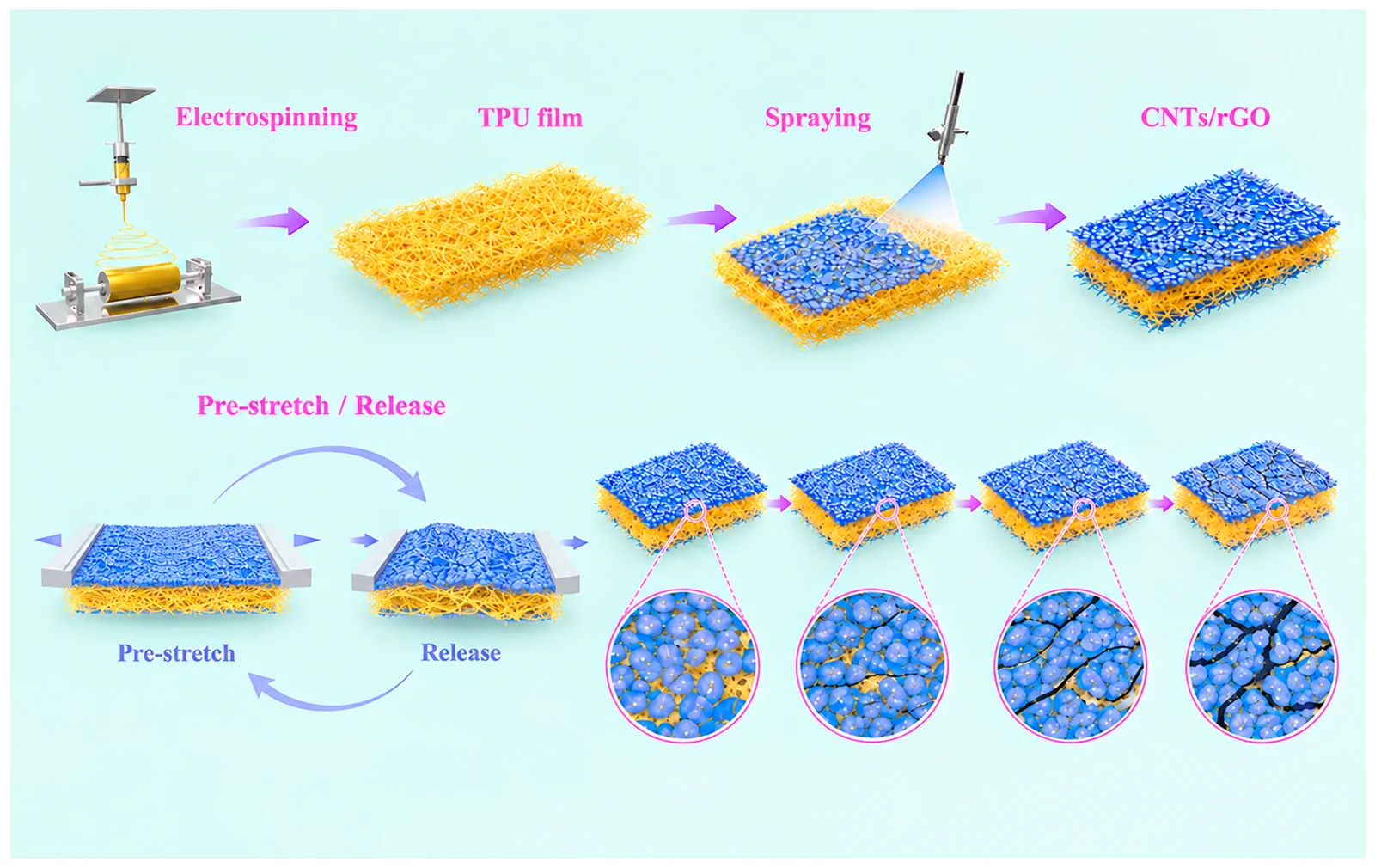
Illustration of the preparation process of CCRTS conductive nanocomposite materials.

As illustrated in Figure 1, flexible TPU nanofiber membranes were first fabricated by electrospinning. A CNTs/rGO hybrid conductive ink was subsequently deposited onto both surfaces of the nanofiber membranes using a high-pressure airbrush, followed by controlled pre-stretching to generate the dual-microcrack conductive architecture and yield the CCRTS. To optimize the conductive network, the effect of spray-coating cycles on the electrical conductivity of the composite membranes was systematically investigated (Figure S1). With increasing spray cycles from 2 to 8, the resistivity decreased markedly from 800 to 75 Ω·cm. Beyond six spray cycles, the reduction in resistivity became negligible, indicating that a highly interconnected conductive network had been established. Accordingly, six spray cycles were adopted for all subsequent experiments and characterizations.

### 3.2. Microscopic Morphological Characterization of CCRTS

**Figure 2:**
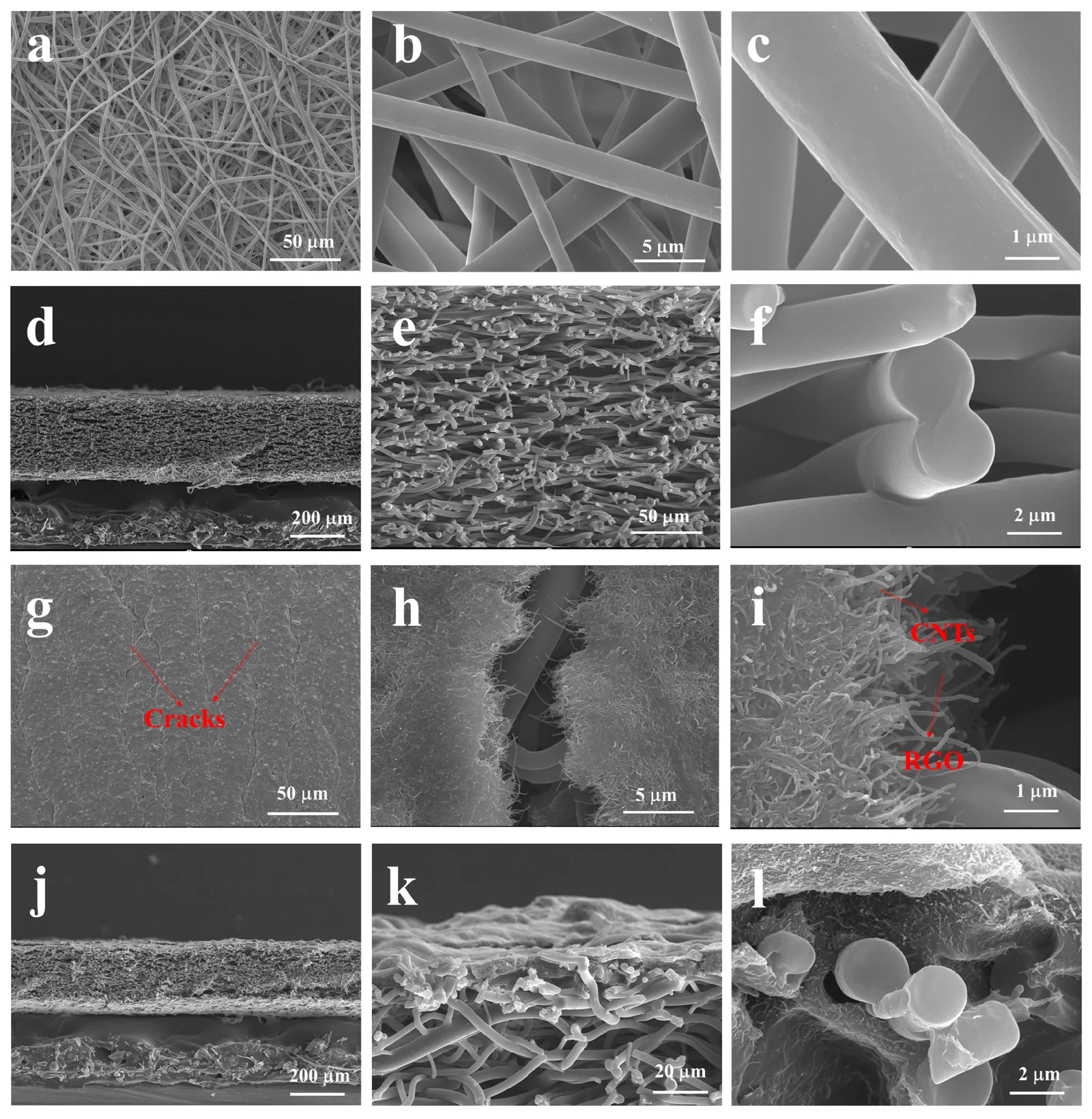
Morphological structure. (**a**–**c**) SEM images of the electrospun TPU nanofiber membrane at different magnifications. (**d**–**f**) Cross-sectional SEM images of the electrospun TPU nanofiber membrane at different magnifications. (**g**–**i**) SEM images of the CCRTS at different magnifications. (**j**–**l**) Cross-sectional SEM images of the CCRTS at different magnifications.

The microscopic morphology of the electrospun TPU nanofiber membrane was first characterized by scanning electron microscopy (SEM). As shown in Figure 2a–c, the pristine TPU membrane exhibits a randomly interconnected fibrous architecture with relatively smooth fiber surfaces and an average fiber diameter of approximately 2 μm. Such a highly porous fibrous network provides a large specific surface area and abundant interfacial sites for the subsequent deposition and anchoring of conductive nanomaterials. The cross-sectional SEM image (Figure 2d) reveals a membrane thickness of approximately 220 μm. Notably, the electrospinning process also generates abundant inter-fiber junctions within the membrane. Because solvent evaporation is not completely achieved when the as-spun fibers are deposited onto the collector, adjacent fibers can partially adhere upon contact, resulting in numerous interconnected junctions throughout the fibrous network (Figure 2e,f). These junctions enhance inter-fiber interactions and promote structural integrity of the membrane, providing an interconnected mechanical framework for subsequent conductive-network construction and deformation-induced microcrack evolution.

Following the pre-stretching treatment, abundant microcracks are generated throughout the CNTs/rGO conductive layer of the composite nanofiber membrane (Figure 2g). This microcrack formation primarily originates from the mechanical mismatch between the relatively rigid conductive layer and the highly compliant TPU substrate, which induces localized strain concentration and interfacial crack evolution during deformation. As shown in Figure 2h, the resulting microcracks exhibit an average width of approximately 3 μm. Notably, the high-aspect-ratio CNTs undergo partial alignment along the stretching direction during pre-stretching, while some CNTs remain interconnected across the crack gaps (Figure 2i). Such crack-spanning conductive bridges preserve partial electrical connectivity while allowing substantial variation in the effective conductive pathways upon subsequent deformation, thereby contributing to the broad strain responsiveness of the sensor.

The cross-sectional morphology of the CNTs/rGO/TPU composite membrane is presented in Figure 2j–l. The conductive ink is not confined to the outer surface but penetrates deeply into the porous fibrous matrix, forming an interpenetrating conductive architecture throughout the membrane. This infiltration is facilitated by the intrinsic porosity of the electrospun TPU network and the high-pressure spray-coating process, which promotes efficient transport of the conductive ink into the inter-fiber voids. The resulting interfacial interlocking between the conductive fillers and TPU fibers strengthens the coupling between the conductive network and polymer substrate, thereby suppressing interfacial instability and improving the reproducibility of the strain-induced resistance response.

Mechanical compliance is essential for flexible strain sensors intended for large-deformation and wearable applications. As shown in Figure S2a,b, the CCRTS withstands substantial tensile deformation without apparent structural failure, demonstrating its ability to accommodate large mechanical strains. Meanwhile, its ultralight and highly compliant nature is further evidenced by its ability to be placed directly onto delicate flower petals without inducing observable bending or damage (Figure S2c). These characteristics, together with the interconnected fibrous architecture, provide a mechanically compliant platform for reliable strain transduction over a broad deformation range.

### 3.3. The Strain-Resistance Response Behavior of CCRTS

**Figure 3:**
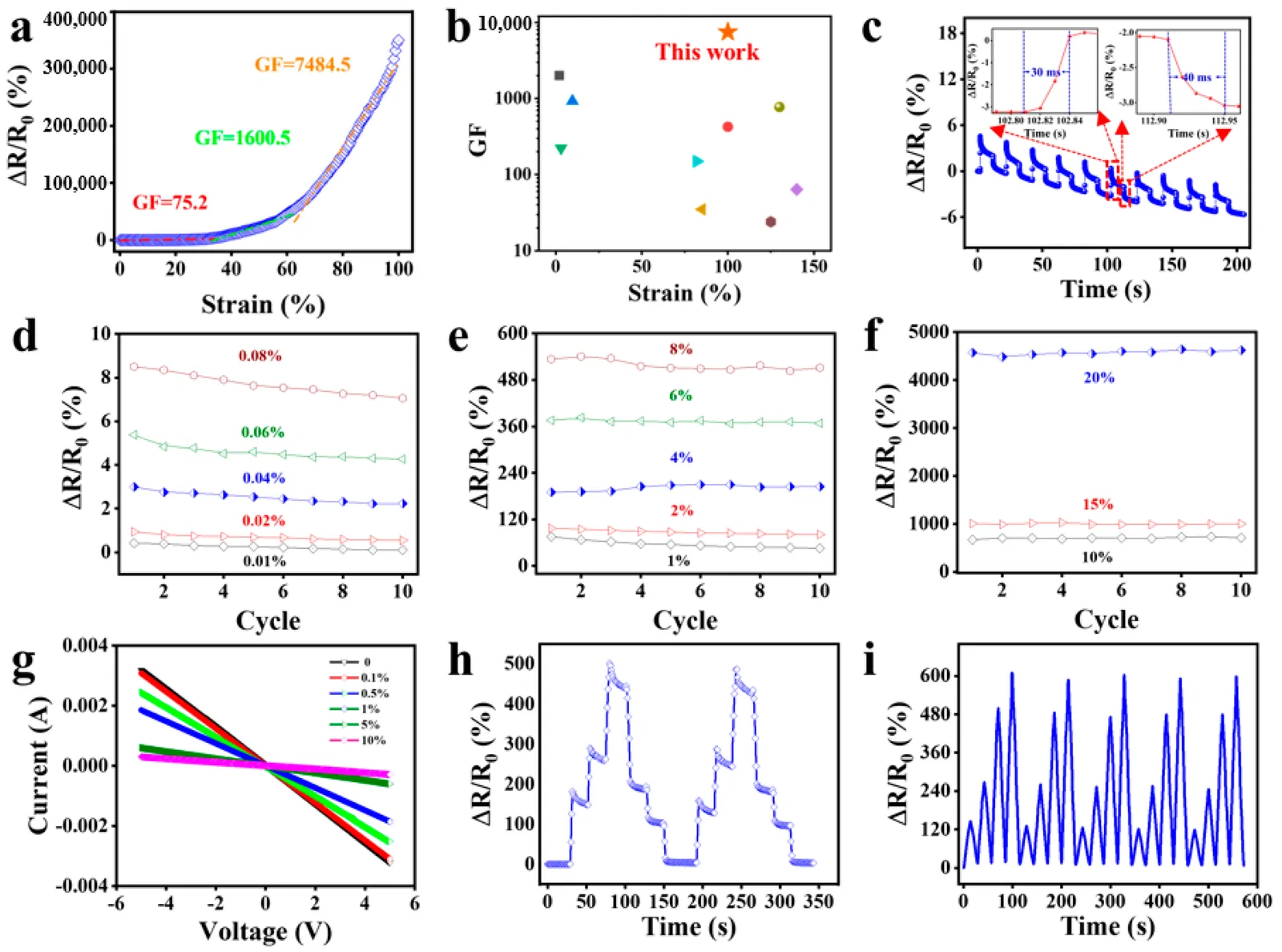
(**a**) Relative resistance change (Δ*R*/*R*_0_) as a function of applied strain for the CCRTS. (**b**) Comparison of the sensing performance of the CCRTS with previously reported flexible strain sensors. (**c**) Dynamic response and response/recovery times of the CCRTS. Maximum Δ*R*/*R*_0_ values under ultralow (**d**), small (**e**), and large (**f**) tensile strains. (**g**) I–V curves of the CCRTS under different tensile strains. (**h**) Relative resistance response under stepped strains of 0–5%. (**i**) Cyclic sensing responses under stepped strains.

The electromechanical response of the CCRTS was subsequently investigated. As shown in Figure 3a, the sensor maintains a stable and distinguishable resistance response over a strain range of up to 100%. The relative resistance change (Δ*R*/*R*_0_) increases nonlinearly with applied strain, reflecting the progressive evolution of the microcracked conductive network. The corresponding gauge factors (GFs) are calculated to be 75.2, 1600.5, and 7484.5 within the strain intervals of 0–32%, 32–60%, and 60–100%, respectively. The stepwise increase in GF with strain indicates that progressive crack opening and conductive-pathway disruption increasingly amplify the resistance variation at higher deformation levels. A comparison of the sensing range and GF with previously reported flexible strain sensors is presented in Figure 3b [37,38,39,40,41,42,43,44,45]. The CCRTS occupies a favorable region combining a broad working range with high sensitivity, highlighting the effectiveness of the dual-microcrack architecture in reconciling the intrinsic sensitivity-range trade-off of conventional strain sensors. Response and recovery kinetics are critical for capturing dynamic mechanical stimuli. To evaluate the dynamic sensing capability of the CCRTS, a small strain of 0.1% was applied at a stretching rate of 1000 mm min^−1^. As shown in Figure 3c, the sensor exhibits a rapid response time of 30 ms and a recovery time of 40 ms (Figure 3c). Such rapid electromechanical kinetics can be attributed to the highly sensitive microcrack-mediated modulation of conductive pathways, enabling the CCRTS to faithfully resolve rapid mechanical perturbations and transient human motions.

The strain-sensing capability of the CCRTS was first evaluated in the ultralow-strain regime. As presented in Figure S3, the sensor produces a distinguishable and repeatable resistance response even at an extremely small tensile strain of 0.01%, demonstrating its ability to resolve subtle mechanical deformation. Notably, the maximum Δ*R*/*R*_0_ gradually decreases during successive stretching–releasing cycles. This behavior is mainly associated with the irreversible rearrangement and in-plane orientation of the high-aspect-ratio conductive fillers along the tensile direction. During cyclic deformation, CNTs and rGO fragments located around the microcracks can partially reconnect and reconstruct conductive pathways upon unloading, thereby reducing the extent of conductive-network disruption in subsequent cycles. Nevertheless, the sensor maintains distinguishable responses at different ultralow strain levels. Statistical analysis of the maximum sensing signals obtained from ten consecutive cycles (Figure 3d) further confirms the reliable discrimination and good repeatability of the CCRTS toward subtle deformation. The sensing behavior was subsequently investigated under small tensile strains of 1–8%. As shown in Figure S4 and Figure 3e, both the signal amplitude and response stability increase progressively with applied strain. At relatively small deformation, the CNTs spanning the microcrack gaps can sustain partial electrical connectivity and facilitate conductive-pathway reconstruction. With increasing strain, however, these crack-bridging CNTs are progressively separated or disrupted, resulting in a rapid reduction in the number of effective conductive contacts and consequently a pronounced increase in electrical resistance. This strain-dependent transition from conductive-pathway preservation to progressive disconnection is responsible for the enhanced signal amplification of the CCRTS. The electromechanical response under relatively large tensile deformation was further examined. As shown in Figure S5 and Figure 3f, the CCRTS exhibits highly repeatable resistance responses under cyclic strains of 10%, 15%, and 20%, with negligible hysteresis. The low hysteresis indicates efficient reversibility of the conductive-network evolution during loading and unloading, while the stable signal output demonstrates that the microcrack architecture can accommodate repeated large deformation without substantial structural degradation. These characteristics provide a reliable basis for monitoring large-amplitude biomechanical motions.

The electrical transport characteristics of the CCRTS were further examined by recording the current–voltage (I–V) relationship under different tensile strains. As shown in Figure 3g, the I–V curves exhibit an approximately linear relationship over the investigated strain range of 0–10%, indicating ohmic conduction within the hybrid conductive network. The preserved linearity under deformation suggests that strain primarily regulates the effective connectivity and contact density of the conductive network rather than introducing substantial changes in the fundamental charge-transport mechanism. This stable electrical transport behavior is essential for reliable resistive strain transduction.

To further evaluate the capability of the CCRTS to distinguish different deformation amplitudes, stepped tensile strains ranging from 0 to 5% were sequentially applied. As shown in Figure 3h, the resistance exhibits discrete and distinguishable changes corresponding to each strain level. A slight resistance relaxation is observed during the strain-holding period, which can be attributed to the viscoelastic stress relaxation of the TPU matrix and the accompanying gradual rearrangement of the conductive network. Importantly, the sensor retains clear stepwise signal characteristics despite this relaxation. Five consecutive stepped-strain cycles from 1% to 5% were subsequently performed (Figure 3i). The highly consistent resistance peaks and near-complete recovery toward the initial resistance after unloading demonstrate the excellent reversibility and repeatability of the CCRTS. Beyond tensile deformation, the strain-sensing behavior of the CCRTS under bending was further investigated. Unlike uniaxial stretching, bending generates spatially heterogeneous deformation across the sensor thickness, leading to the simultaneous evolution of conductive pathways under tensile and compressive stresses. During bending, the outer surface undergoes tensile deformation, which promotes microcrack opening and disrupts conductive contacts, whereas the inner surface experiences compression, which narrows the crack gaps and facilitates conductive-pathway compaction and reconstruction. Consequently, the electrical response is governed by the competition between conductive-network disruption and reconstruction.

As shown in Figure S6a, the relative resistance initially increases with increasing bending deformation, indicating that crack opening and conductive-pathway disruption dominate at relatively small bending angles. With further deformation, the resistance decreases rapidly, suggesting that conductive-pathway reconstruction and network compaction become increasingly dominant. Upon removal of the bending deformation, the resistance nearly returns to its initial value, demonstrating the reversible evolution of the microcracked conductive network. The CCRTS also maintains stable and reproducible responses under cyclic bending at a bending strain of 10% and a bending/releasing rate of 10 mm min^−1^ (Figure S6b). Similar sensing stability is retained when the bending strain is increased to 20% (Figure S6c). Furthermore, negligible signal degradation is observed over 100 consecutive bending–releasing cycles (Figure S6d), confirming the excellent reversibility and mechanical durability of the dual-microcrack conductive architecture.

Collectively, these results reveal that the sensing mechanism of the CCRTS is governed by dynamic regulation of conductive-pathway connectivity, involving crack opening, conductive-contact separation, filler rearrangement, and pathway reconstruction. The resulting multiscale and reversible evolution of the conductive network enables the sensor to resolve deformation ranging from ultralow strains to large tensile and bending motions, providing a robust structural basis for high-performance flexible strain sensing.

### 3.4. Strain-Sensing Mechanism of CCRTS

**Figure 4:**
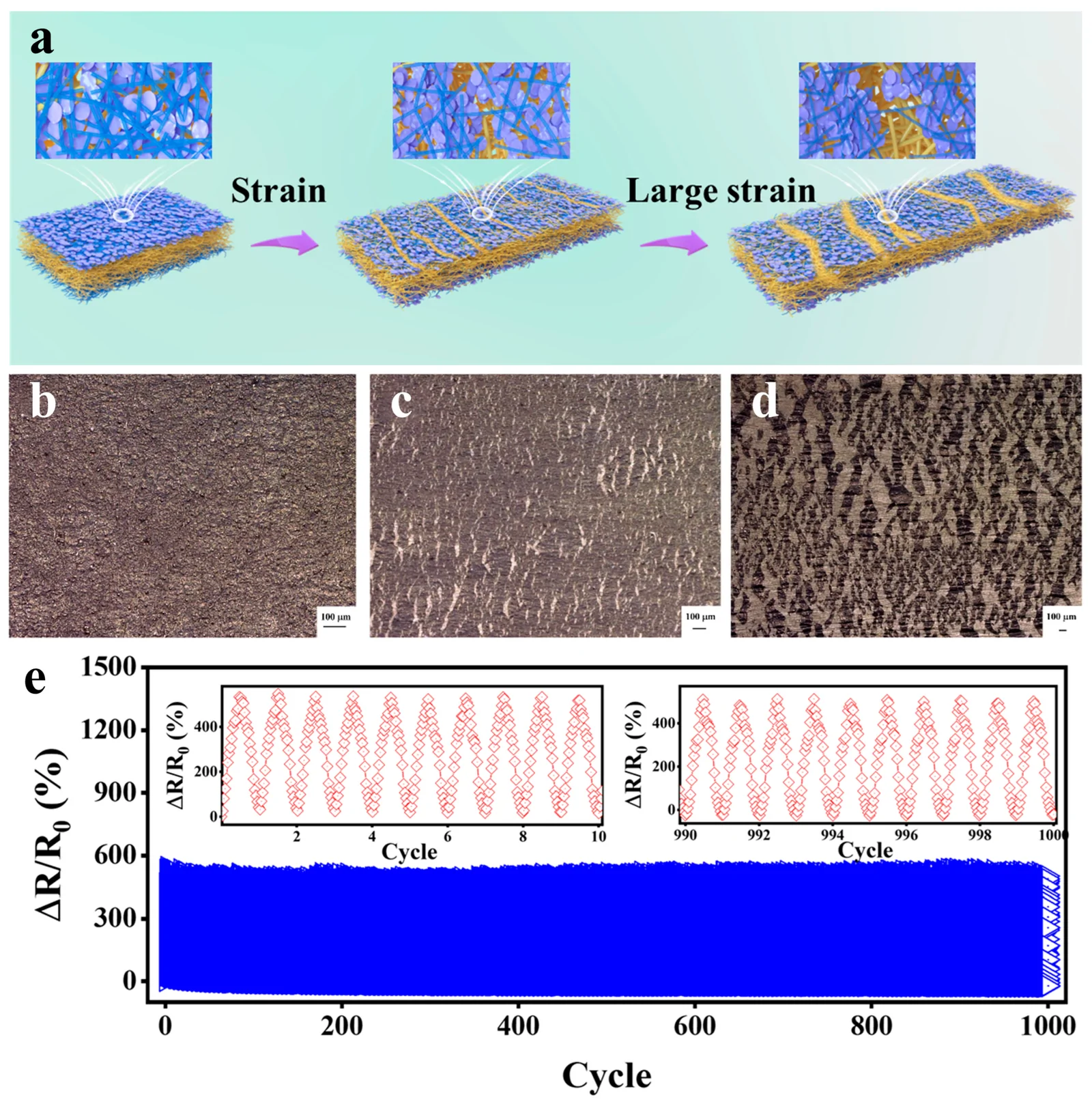
(**a**) Strain-resistance response mechanism diagram of CCRTS. (**b**–**d**) The optical images of cracks formed during the stretching process of CCRTS. (**e**) The cyclic durability test of CCRTS.

Figure 4a schematically illustrates the strain-resistance transduction mechanism of the CCRTS. The pre-stretching process generates a dense microcrack network within the CNTs/rGO conductive layer as a consequence of the mechanical mismatch between the rigid conductive layer and the compliant TPU substrate. Upon tensile deformation, the evolution of these microcracks continuously regulates the connectivity of the conductive network, thereby converting mechanical strain into amplified electrical resistance changes. At relatively small strains, rGO nanosheets dominate the electromechanical response owing to their intrinsically high sensitivity to interfacial separation. As the microcracks progressively open, the contact area and tunneling pathways between adjacent rGO nanosheets decrease rapidly, resulting in a pronounced increase in electrical resistance. With further deformation, the nucleation and propagation of additional microcracks progressively disrupt the conductive network, leading to an increasingly amplified resistance response, consistent with the crack evolution observed in the optical images (Figure 4b-d). Upon tensile loading, evolving microcracks may modulate rGO tunneling barriers while interpenetrating CNTs potentially act as crack-spanning conductive bridges; strain-induced rearrangement of the hybrid CNTs/rGO network cooperates with crack development to produce the observed segmented gauge-factor response. In contrast, the high-aspect-ratio CNTs form conductive bridges across adjacent rGO domains and crack gaps, preserving residual electrical connectivity even after local network disruption. This complementary role of rGO and CNTs enables the conductive network to simultaneously retain high strain sensitivity and withstand substantial deformation. Thus, the strain-dependent competition between conductive-pathway disruption and CNT-mediated pathway bridging provides the fundamental structural origin of the broad sensing range and high sensitivity of the CCRTS.

Long-term mechanical stability is essential for reliable operation of wearable strain sensors. To evaluate the fatigue resistance and sensing reproducibility of the CCRTS, 1000 consecutive stretching–releasing cycles were conducted at a fixed strain of 5% and a stretching rate of 5 mm min^−1^. As shown in Figure 4e, the sensor maintains highly consistent resistance profiles throughout the repeated deformation cycles, with no appreciable signal degradation. The excellent cyclic stability can be attributed to the robust integration between the infiltrated conductive network and the electrospun TPU fibrous matrix, together with the reversible evolution of the microcrack architecture. These results confirm the mechanical robustness and operational reliability of the CCRTS under repeated deformation.

### 3.5. The Application of CCRTS in the Collection of Human Biological Signals

**Figure 5:**
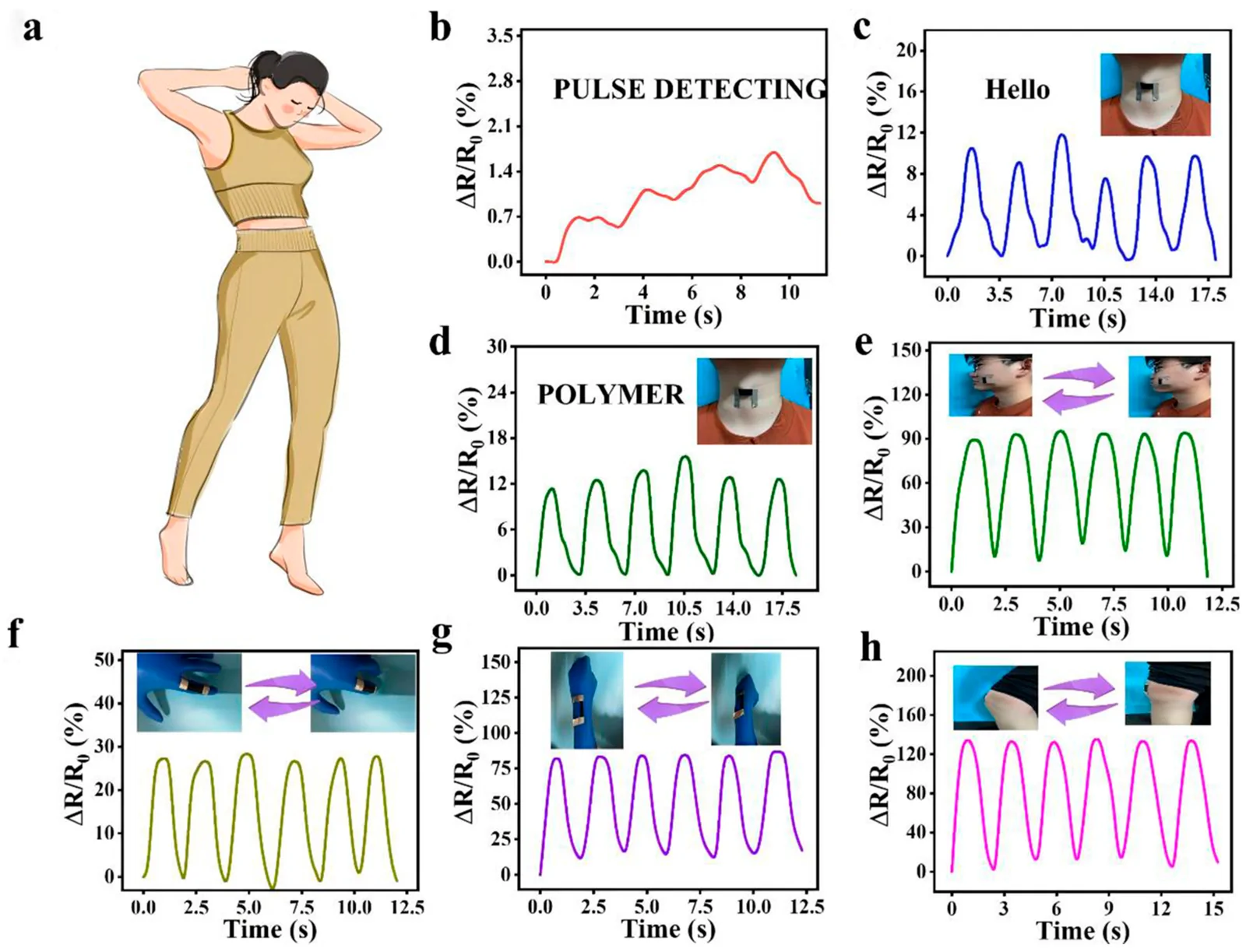
Application of human biological signal collection. (**a**) The application potential of CCRTS in human biological signals the collection. The Δ*R*/*R*_0_ curves of CCRTS in response to pulse (**b**), vibration (**c**,**d**) and mouth blowing (**e**), finger bending (**f**), wrist bending (**g**), knee bending (**h**).

Benefiting from its superior stretchability, high sensitivity, broad sensing range, rapid response, ultralow detection limit, and excellent recoverability and repeatability, the CCRTS can serve as a flexible wearable device for monitoring both subtle and vigorous human motions (Figure 5a). Owing to its high sensitivity, the CCRTS can precisely capture weak human pulse signals (Figure 5b), demonstrating great potential for health monitoring and medical applications. When attached to the human larynx, the sensor effectively distinguishes vocal movements corresponding to different pronunciations, such as “HELLO” and “POLYMER” (Figure 5c,d), highlighting its potential for laryngeal rehabilitation. For facial motion detection, the sensor adhered to the cheek undergoes deformation during cheek bulging, increasing the relative resistance of the CCRTS; the resistance rapidly recovers to its initial state upon release, enabling effective monitoring of facial expressions (Figure 5e). Owing to its broad sensing range, the CCRTS is also suitable for monitoring large-scale human joint movements. When attached to the index finger (Figure 5f), the sensor accurately records repeated bending–straightening motions. The relative resistance increases with increasing finger bending and rapidly returns to its initial level after unloading, demonstrating excellent recoverability and sensing stability. Similarly, the CCRTS can effectively monitor large-amplitude human motions, including wrist bending (Figure 5g) and knee flexion (Figure 5h). Wearable human-motion measurements serve as proof-of-concept tests to validate the fundamental on-body strain-sensing performance of the sensor, revealing its high application potential toward practical medical and health-monitoring scenarios.

## 4. Conclusions

In summary, a flexible strain sensor (CCRTS) featuring a dual-microcrack conductive network is developed by integrating electrospun TPU nanofibers, CNTs/rGO hybrid conductive networks, and controlled pre-stretching. The complementary 1D CNTs and 2D rGO networks provide sustained conductive pathways under deformation, while the modulus mismatch between the conductive layer and TPU substrate induces strain-responsive dual-microcracks, thereby amplifying resistance variation and enhancing strain sensitivity. Consequently, the CCRTS achieves a broad sensing range of up to 100% strain, a maximum GF of 7484.5, rapid response/recovery times of 30/40 ms, an ultralow detection limit of 0.01% strain, and excellent cyclic stability. The sensor further enables reliable monitoring of both subtle physiological signals, including pulse, vocalization, and facial motion, and large-scale human movements such as finger, wrist, and knee bending. This work highlights the synergistic regulation of conductive-network connectivity and microcrack evolution as an effective strategy for overcoming the sensitivity–range trade-off, providing a versatile design platform for high-performance flexible strain sensors.

## Data Availability

The original contributions presented in this study are included in the article/Supplementary Materials. Further inquiries can be directed to the corresponding author.

## Supplementary Materials

The following supporting information can be downloaded at: https://www.mdpi.com/article/10.3390/nano16191234/s1.
