# Supplementary material for: Flexible Strain Sensor Based on TPU/CNTs/rGO Conductive Membrane with Dual-Microcrack Structure for High-Performance Wearable Sensing
Source: Nanomaterials (Basel). 2026 Sep 30;16(19):1234. doi: 10.3390/nano16191234 (PMC13666651; doi:10.3390/nano16191234)
Supplement: Supplementary file 1 [file nanomaterials-16-01234-s001.zip › nanomaterials-4584968-supplementary.pdf]

Supporting Information

Flexible Strain Sensor Based on TPU/CNTs/rGO Conductive Membrane with Dual-Microcrack Structure for High-Performance Wearable Sensing

Jinlong Chen [1,2], Binghao Xing [1], Yuexin Wang [1], Huimin Guo [1], Chenrui Zhu [1], Weihua Wang [1], Junfang Shen [1,2], Zhaowu Wang [1], Pengbo Zhao [2], Qian Sun [1,2], Derong Zhu [1,2], Zhiyu Min [2,3,]* and Wei Zhai [4,]*

1 School of Intelligent Manufacturing, Luoyang Institute of Science and Technology, Luoyang 471023, China; jlchen@lit.edu.cn (J.C.)

2 Henan Key Laboratory of Green Building Materials Manufacturing and Intelligent Equipment, Luoyang Institute of Science and Technology, Luoyang 471023, China

3 Henan Mechanical and Electrical Vocational College, Zhengzhou 451192, China

4 Key Laboratory of Materials Processing and Mold of Ministry of Education, School of Materials Science and Engineering, Zhengzhou University, Zhengzhou 450001, China

* Correspondence: 200900300185@lit.edu.cn (Z.M.); weizhai@zzu.edu.cn (W.Z.)

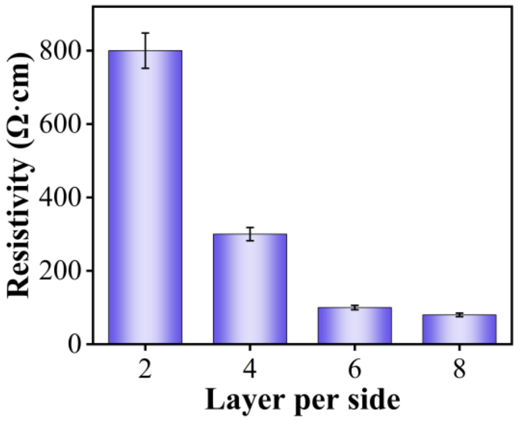

Figure. S1. The influence of different spraying frequencies on the conductivity of the composite material.

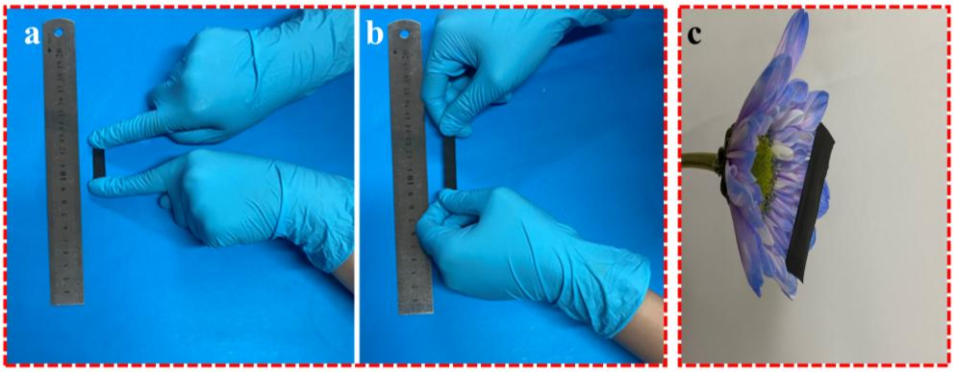

Figure S2. The excellent mechanical properties of CCRTS of (a) Elasticity, (b) Lightweight.

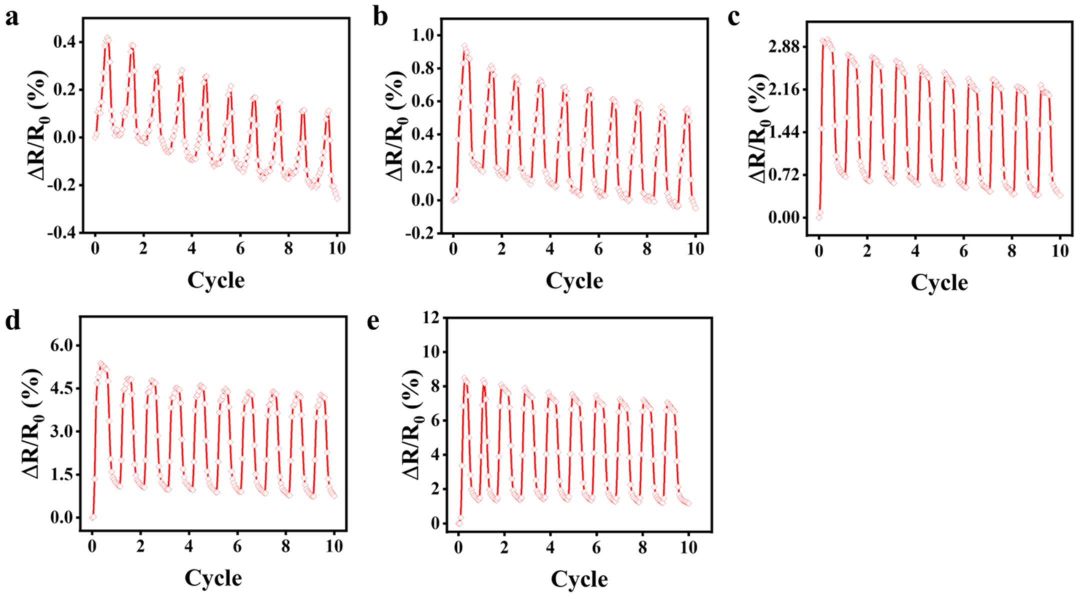

Figure S3. The 10 repetitive tensile-recovery sensing performances of CCRTS under different extremely small tensile strains (a) 0.01%, (b) 0.02%, (c) 0.04%, (d) 0.06%, (e) 0.08%.

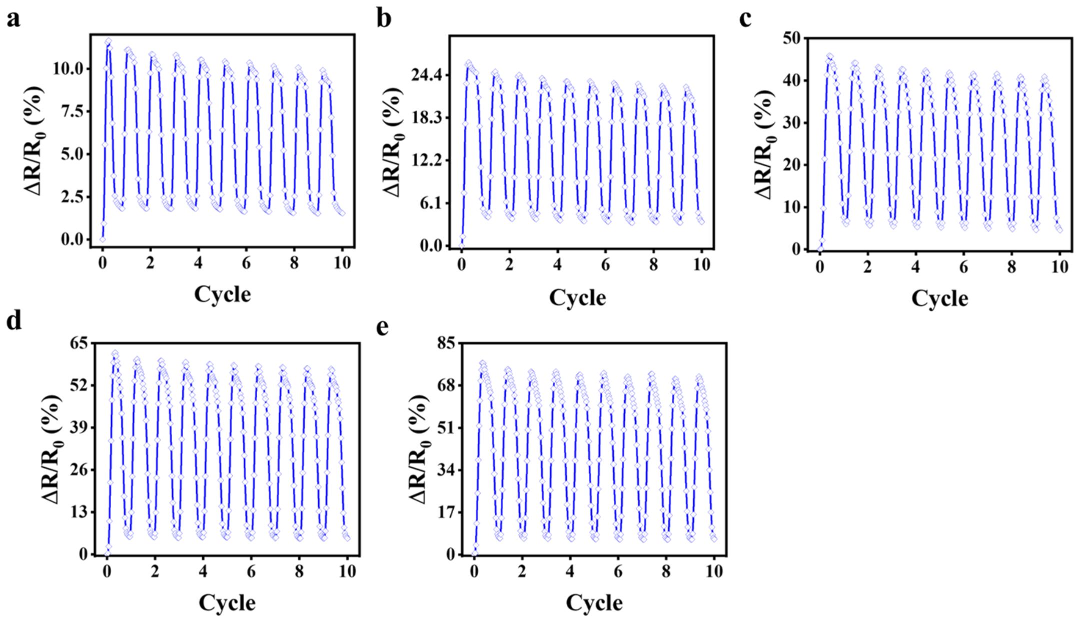

Figure S4. The 10 repetitive tensile-recovery sensing performances of CCRTS under different extremely small tensile strains (a) 1%, (b) 2%, (c) 4%, (d) 6%, (e) 8%.

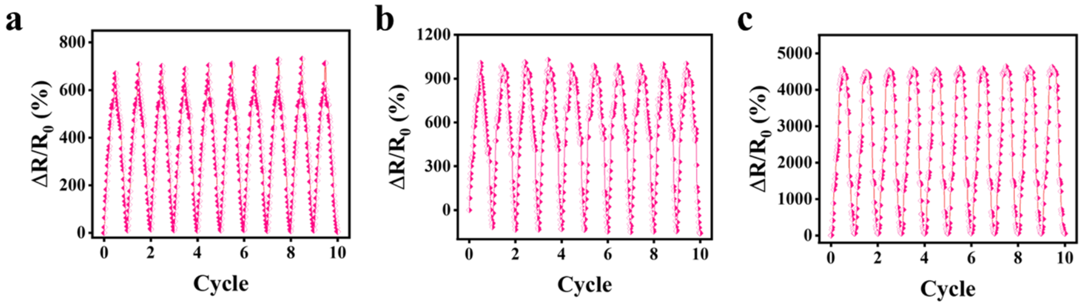

Figure S5. The 10 repetitive tensile-recovery sensing performances of CCRTS under different extremely small tensile strains (a) 10%, (b) 15%, (c) 20%.

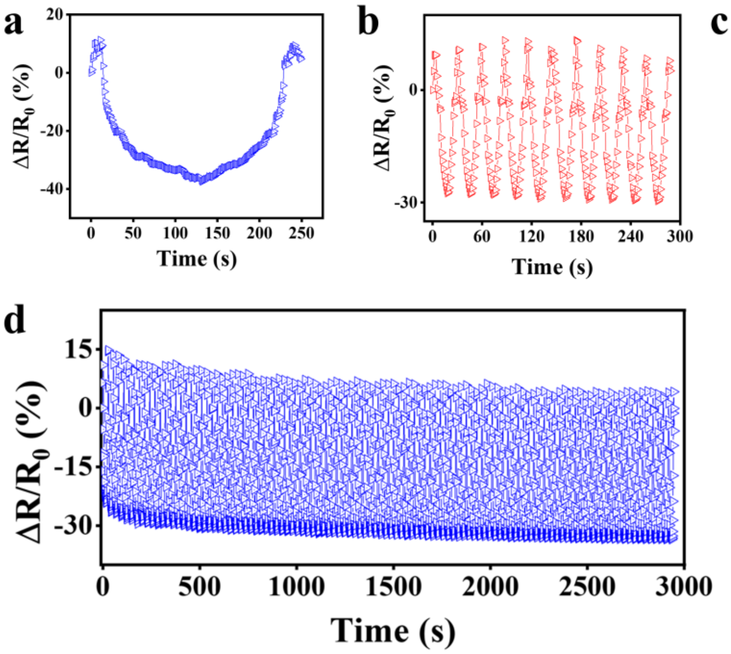

Figure S6. (a) The bending resistance response performance of CCRTS. (b-c) The strain-resistance response performance of CCRTS under different bending strains. (d) The long-term cyclic durability of CCRTS under bending deformation.
